# Measuring Granger Causality between Cortical Regions from Voxelwise fMRI BOLD Signals with LASSO

**DOI:** 10.1371/journal.pcbi.1002513

**Published:** 2012-05-24

**Authors:** Wei Tang, Steven L. Bressler, Chad M. Sylvester, Gordon L. Shulman, Maurizio Corbetta

**Affiliations:** 1 Center for Complex Systems and Brain Sciences, Florida Atlantic University, Boca Raton, Florida, United States of America; 2 Department of Psychology, Florida Atlantic University, Boca Raton, Florida, United States of America; 3 Department of Radiology, Washington University School of Medicine, St. Louis, Missouri, United States of America; 4 Department of Neurology, Washington University School of Medicine, St. Louis, Missouri, United States of America; 5 Department of Neurobiology, Washington University School of Medicine, St. Louis, Missouri, United States of America; Indiana University, United States of America

## Abstract

Functional brain network studies using the Blood Oxygen-Level Dependent (BOLD) signal from functional Magnetic Resonance Imaging (fMRI) are becoming increasingly prevalent in research on the neural basis of human cognition. An important problem in functional brain network analysis is to understand directed functional interactions between brain regions during cognitive performance. This problem has important implications for understanding top-down influences from frontal and parietal control regions to visual occipital cortex in visuospatial attention, the goal motivating the present study. A common approach to measuring directed functional interactions between two brain regions is to first create nodal signals by averaging the BOLD signals of all the voxels in each region, and to then measure directed functional interactions between the nodal signals. Another approach, that avoids averaging, is to measure directed functional interactions between all pairwise combinations of voxels in the two regions. Here we employ an alternative approach that avoids the drawbacks of both averaging and pairwise voxel measures. In this approach, we first use the Least Absolute Shrinkage Selection Operator (LASSO) to pre-select voxels for analysis, then compute a Multivariate Vector AutoRegressive (MVAR) model from the time series of the selected voxels, and finally compute summary Granger Causality (GC) statistics from the model to represent directed interregional interactions. We demonstrate the effectiveness of this approach on both simulated and empirical fMRI data. We also show that averaging regional BOLD activity to create a nodal signal may lead to biased GC estimation of directed interregional interactions. The approach presented here makes it feasible to compute GC between brain regions without the need for averaging. Our results suggest that in the analysis of functional brain networks, careful consideration must be given to the way that network nodes and edges are defined because those definitions may have important implications for the validity of the analysis.

## Author Summary

Modern cognitive neuroscience views cognition in terms of brain network function. A network is a physical system of nodes connected to each other by edges. From the network perspective, cognitive function depends on activity patterns involving the nodes and edges of functional brain networks. It is important then, to appropriately define the nodes and edges of functional brain networks in order to understand cognition. In this study we consider the nodes of functional brain networks to be brain regions, and demonstrate a method that effectively measures the edge pattern between regions with a technique called Granger Causality. Our method is made possible by the utilization of recent advances from the field of statistics. Our approach is generally applicable to functional brain network analysis and contributes to the understanding of network properties of the brain.

## Introduction

The modern understanding of human cognition relies heavily on the concept of large-scale functional brain networks, and large-scale functional network analysis of Blood-Oxygenation-Level-Dependent (BOLD) signals from functional Magnetic Resonance Imaging (fMRI) is playing an increasingly important role in cognitive neuroscience [1]. From this perspective, knowledge of cognition may be obtained from BOLD signals by identification of the nodes and edges of large-scale functional brain networks. An important unresolved question remaining in the field, however, is how best to define the nodes and edges of large-scale functional brain networks.

A node is typically represented in brain network studies of fMRI BOLD activity as a lumped Region Of Interest (ROI), formed by averaging the BOLD signals of all the ROI's voxels [2]–[6]. This collapse of the ROI by averaging has the benefit of reducing the dimensionality of analysis, but rests on the twin assumptions: (1) that the BOLD activity of an ROI is homogeneous over all its voxels; and (2) that the functional interactions (connectivity) between the voxels of an ROI with those in other ROIs is also homogeneous. If these homogeneity assumptions are not true, edge measurements computed from ROI-averaged BOLD signals may be erroneous since averaging may distort the time series information.

Here we present a novel procedure for the analysis of directed interregional functional interactions that is based on the BOLD activity of the individual voxels of ROIs and the Granger Causality (GC) measure of directed interaction between voxels. GC tests whether the prediction of the present value of one time series by its own past values can be significantly improved by including past values of another time series in the prediction. If so, the second time series is said to Granger cause the first, and the degree of significance of the improvement may be taken as the strength of GC [7]. The GC measure is typically implemented by AutoRegressive (AR) modeling [8] and has been shown to be a powerful and flexible tool for measuring the predictability of one neural time series from another [9]–[14]. It has advantages as an edge measure over the typically utilized correlation: first, it provides the strength of functional interaction between time series in both directions, as opposed to a single non-directional strength; second, its grounding in prediction allows stronger statements to be made about functional interactions than does simple correlation. The use of GC to measure directed interactions in the brain from fMRI BOLD data has received intense scrutiny in recent years, with some arguing in its favor [15]–[18] and others opposed to it [19]–[23]. In the present work, we focus on improving the application of GC analysis to fMRI BOLD data in order to better understand the role of top-down influences in visuospatial attention.

Previous evidence from GC analysis of fMRI BOLD data argues against the assumption of homogeneous interregional functional interactions, and thus suggests that averaging BOLD signals prior to edge measurement may not be appropriate. Bressler et al. [24] found that GC between ROIs varies considerably across voxel pairs, with the distribution of GC values being highly skewed and only a small fraction of values in the tail of the distribution being significantly different from zero. These results indicate that GC is heterogeneous across voxel pairs, suggesting that the investigation of functional interactions between ROIs should take into account the interactions of all the voxels within the ROIs.

**Figure 1:**
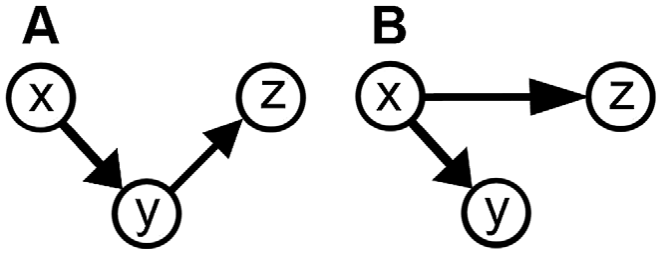
Simple driving patterns that can lead to spurious identification of significant Granger Causality. A) Sequential driving pattern, where voxel *x* drives voxel *y*, which in turn drives voxel *z*. GC from *x* to *z* may be spuriously identified as being significant. B) Differentially delayed driving, where voxel x drives voxel *y* with shorter delay and *z* with longer delay. GC from *y* to *z* may be spuriously identified as being significant. Modified from [26].

An approach to the problem of heterogeneous functional interaction between ROIs is to compute the distribution of GC values using a bivariate AR model for each pairwise combination of voxels in the ROIs. This pairwise-GC approach, followed by Bressler et al. [24], not only avoids the possible pitfalls of averaging, but also makes feasible the separate measurement of GC density and strength between ROIs, two factors that are conflated by averaging. Thus, deriving a summary GC statistic between ROIs from the distribution of GC values across all voxel-voxel pairs may be statistically more informative than simply setting it to the GC between across-voxel averages. There is a further problem, however, with the pairwise-GC measure: some GC values may be identified as being significant when actually they are not. This problem arises, for example, if one voxel (*x*) ‘drives’ a second voxel (*y*), while voxel *y* ‘drives’ a third voxel (*z*), without there being a ‘drive’ from voxel *x* to voxel *z* (Figure 1A). In this case, the GC from voxel *x* to voxel *z* may be spuriously identified as being significant. As another example, the problem also occurs if voxel *x* ‘drives’ both voxels *y* and *z* with different delays, without there being a ‘drive’ from *y* to *z* (Figure 1B). In this case, the GC from *y* to *z* may be spuriously identified as being significant. Since *x*, *y*, and *z* may be in the same or different ROIs, one point these examples make clear is that the GC within ROIs should be taken into account in order to reduce the possibility of spuriously identifying GCs as being significant. A second point is that improvement in the GC computation may be possible by using a method that can mitigate the problem of spurious GC significance.

Our approach to the problem of spurious GC significance rests on the concept of conditional GC [25]–[27]. Conditional GC analysis tests for a significant GC from one time series to a second with the effect of a third time series removed. By this procedure, it is possible to determine whether a significant GC measured between two time series is attributable to the third time series. In this paper, we utilize the conditional GC concept for ROI-level analysis in an approach that essentially measures the GC between any pair of voxels in two ROIs conditional on all the other voxels in the ROIs. This is accomplished by constructing a single Multivariate Vector AutoRegressive (MVAR) model from the voxel time series, as opposed to the pairwise-GC method, in which a separate bivariate AR model is constructed for each voxel pair. Use of the MVAR model offers the promise of reducing or eliminating the problem of spurious significant GC identification in the assessment of directed functional interactions from fMRI BOLD signals.

To make use of the MVAR model for ROI-level GC analysis necessitates overcoming one further problem that often occurs in model estimation: the number of available observations (data points) limits the number of parameters (model coefficients) that can accurately be estimated. This problem commonly arises in neurobehavioral studies where the number of data points that can realistically be acquired limits the size of the MVAR model that can be estimated. This limitation can be mitigated, however, if it is assumed that the voxel-voxel functional interactions between ROIs are sparse (i.e., have a low connectivity density) [28]. Under the assumption of sparseness (low connectivity density), the Least Absolute Shrinkage and Selection Operator (LASSO) algorithm [29] is used to pre-select variables for inclusion in the MVAR model, and thus to overcome the problem of a limited number of data points. The LASSO algorithm has previously been tested on numerical experiments [30], gene-network data [31] and simulated and experimental fMRI BOLD data [28], [31]–[33].

This paper presents a novel application of the MVAR model to study voxel-based region-to-region interactions in the brain, particularly long-range, top-down interregional interactions in visuospatial attention. We demonstrate that the LASSO algorithm can be effectively used to pre-select model variables, thereby enabling estimation of the coefficients of a voxel-based MVAR model of two predefined ROIs. The originality of our methods derives from: (1) estimation of the MVAR model for fMRI voxel-level BOLD time series from two ROIs; (2) use of the LASSO algorithm for variable pre-selection prior to MVAR model estimation; (3) use of the General Cross-Validation criterion for determining optimal predictors in the MVAR model from the LASSO algorithm; and (4) creation of two types of summary statistics at the ROI level that represent the separate measurement of density and strength of GC between ROIs.

We report the results of both MVAR model simulations and the application of MVAR model estimation to an empirical fMRI BOLD dataset obtained during a visuospatial attention task [34]. The simulation results demonstrate that voxel-based approaches can better capture the GC between two ROIs than the averaging approach. When LASSO is used to pre-select variables for inclusion in the MVAR model estimation, voxel-based GC summary statistics are more sensitive to coefficient changes in the model than GC values computed from averaged signals. LASSO pre-selection allows MVAR model estimation to fit the simulated data accurately as long as the GC functional connectivity is sparse, i.e. has relatively low density. We also report that construction of the voxel-based GC distribution by pairwise bivariate AR model estimation, instead of by MVAR model estimation with LASSO pre-selection, may yield spuriously significant GC values. The empirical results show that the assumption of sparse GC functional connectivity is realistic, and that LASSO variable pre-selection followed by MVAR model estimation is thus effective, for empirical fMRI BOLD data. Also, the low GC connectivity density observed for this dataset suggests that directed interregional functional interaction in the brain is heterogeneous and that averaging the voxels of an ROI prior to GC connectivity analysis is inappropriate. Furthermore, the observed directional asymmetry, as measured by the GC strength summary statistic, is consistent with current theory on top-down modulation in visuospatial attention.

We conclude that LASSO variable pre-selection and MVAR model estimation can be effectively used to measure Granger Causality between cortical regions from voxelwise fMRI BOLD signals. Through the MVAR model, it is beneficial to analyze all the voxels in an ROI, instead of taking an average over the ROI. In this way, directed interregional functional interactions are captured with less distortion of the information carried in the BOLD time series.

## Results

### Application to simulated data

Simulation MVAR models were created based on Equation 3 (see Methods), and iterated to generate simulated fMRI BOLD time series data for pseudo-voxels in two pseudo-ROIs having fixed sizes (30 pseudo-voxels in X and 50 pseudo-voxels in Y). The innovation process for the simulation model was created by iterative random sampling of a zero-mean normal distribution with 0.1 standard deviation. The predictors were initialized with random values also taken from a zero-mean normal distribution with 0.1 standard deviation. The four coefficient submatrices (B_xx_, B_yx_, B_xy_ and B_yy_) were constructed separately. For each submatrix, some coefficients (*b_ij_*) were randomly set to zero and the rest were randomly drawn from a normal distribution with zero-mean and a specific standard deviation (0.08 for B_xx_ and B_yy_, 0.2 for B_yx_, and 0.1 for B_xy_). For each simulation, 200-point-long time series for each pseudo-voxel were created by model iteration. A total of 56 simulation models were created. The density of model connectivity was systematically increased with increasing model identification number by augmenting the number of voxel pairs connected by non-zero *b* values (Table 1).

**Table 1:** The fraction of non-zero coefficients in each of the 4 submatrices for each of the 56 simulation models.

Sim. ID	B_xx_	B_yy_	B_yx_	B_xy_	Sim. ID	B_xx_	B_yy_	B_yx_	B_xy_
1	0.0656	0.0592	0.0493	0.0420	29	0.1944	0.1768	0.1593	0.1580
2	0.0656	0.0592	0.0473	0.0373	30	0.1944	0.1768	0.1413	0.1473
3	0.0656	0.0592	0.0453	0.0427	31	0.1944	0.1768	0.1373	0.1627
4	0.0656	0.0592	0.0400	0.0433	32	0.1944	0.1768	0.1240	0.1647
5	0.0656	0.0592	0.0427	0.0453	33	0.1944	0.1964	0.1853	0.1500
6	0.0656	0.0592	0.0413	0.0520	34	0.1944	0.1964	0.1707	0.1460
7	0.0656	0.0592	0.0320	0.0580	35	0.2267	0.2160	0.1807	0.1620
8	0.0656	0.0592	0.0340	0.0507	36	0.2267	0.2160	0.2000	0.1920
9	0.0978	0.0984	0.0973	0.0553	37	0.2267	0.2160	0.1800	0.1767
10	0.0978	0.0984	0.0860	0.0727	38	0.2267	0.2160	0.1727	0.2193
11	0.0978	0.0984	0.0827	0.0760	39	0.1944	0.1964	0.1367	0.1860
12	0.0978	0.0984	0.0860	0.0607	40	0.2267	0.2160	0.1687	0.2160
13	0.0978	0.0984	0.0907	0.0680	41	0.2589	0.2552	0.2380	0.2200
14	0.0978	0.0984	0.0713	0.0847	42	0.2267	0.2356	0.2113	0.1947
15	0.0978	0.0984	0.0787	0.0800	43	0.2267	0.2356	0.2213	0.1927
16	0.0978	0.0984	0.0713	0.0907	44	0.2267	0.2356	0.2113	0.2180
17	0.1300	0.1376	0.1233	0.0933	45	0.2589	0.2552	0.2220	0.2360
18	0.1300	0.1376	0.1220	0.1073	46	0.2267	0.2356	0.2027	0.2113
19	0.1300	0.1376	0.1187	0.1107	47	0.2267	0.2356	0.1740	0.2013
20	0.1300	0.1376	0.1300	0.0993	48	0.2589	0.2552	0.1720	0.2613
21	0.1300	0.1376	0.1193	0.1147	49	0.2911	0.2944	0.2847	0.2467
22	0.1300	0.1376	0.0993	0.1213	50	0.2589	0.2552	0.2387	0.1773
23	0.1300	0.1376	0.0980	0.1313	51	0.2911	0.2944	0.2540	0.2680
24	0.1300	0.1376	0.0880	0.1327	52	0.2589	0.2552	0.2373	0.2120
25	0.1944	0.1768	0.1800	0.0920	53	0.2589	0.2552	0.1880	0.2280
26	0.1944	0.1768	0.1727	0.0933	54	0.2911	0.2944	0.2367	0.2947
27	0.1944	0.1768	0.1587	0.1473	55	0.2911	0.2944	0.2053	0.2680
28	0.1944	0.1768	0.1560	0.1500	56	0.2911	0.2944	0.1827	0.2907

The fraction changed over the models from values of approximately 0.05 to values of approximately 0.29, increasing by approximately 0.04 every 8 models.

To verify model validity, we determined that the correlations of the model residuals were low for all 56 models. A representative residuals correlation matrix from one of the simulations is displayed in the Figure S6, showing randomly distributed weak correlation across simulated voxel pairs. We considered the models to be valid based on these observations.

**Figure 2:**
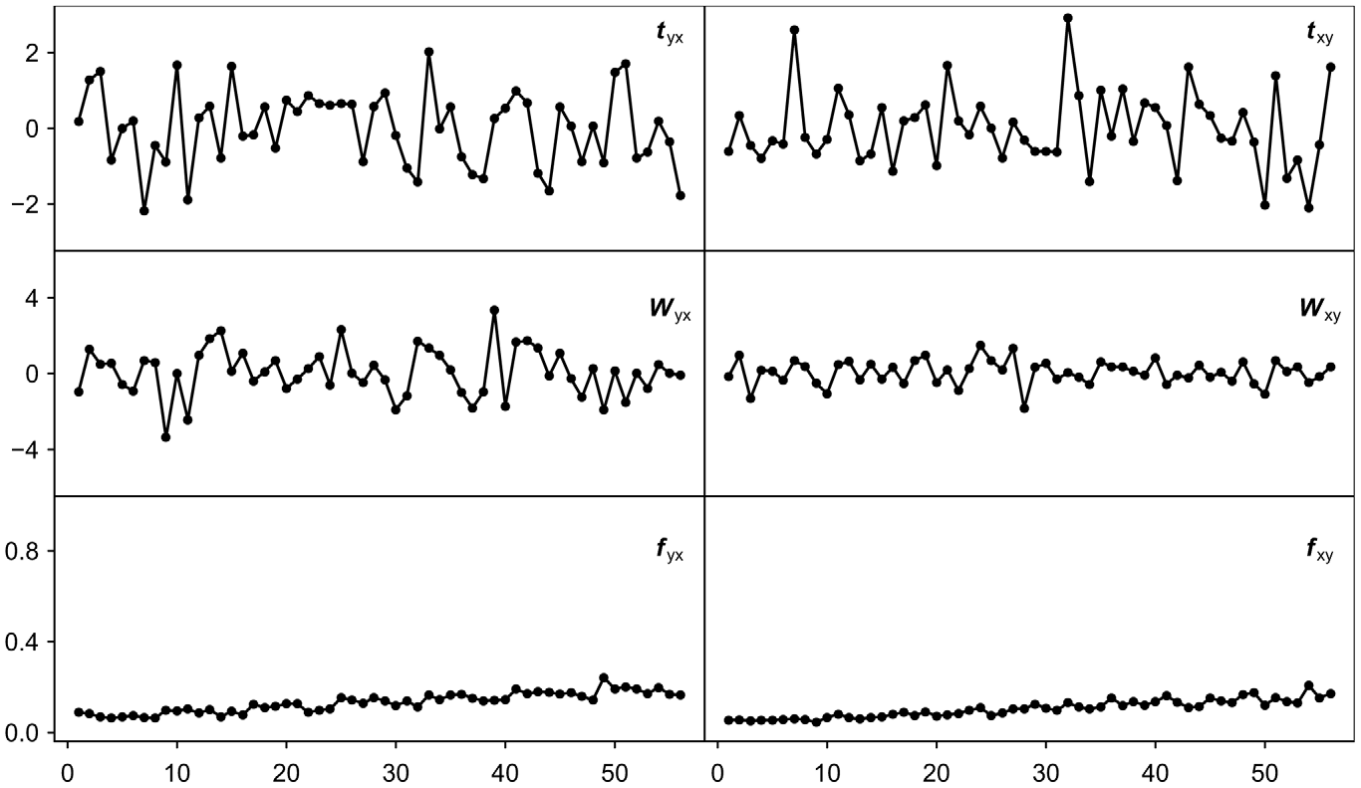
Granger Causality patterns between simulated ROIs. GC was computed from averaged voxel time series as *t*_yx_ and *t*_xy_, and then normalized to *z*-scores, for a range of simulation models (top row). GC was also computed as voxel-based *W* (middle row) and *f* (bottom row) summary statistics computed directly from the parameters of the same simulation models (*b* values normalized to *z*-scores before computing *W*). The horizontal axis labels the 56 simulation models in the order of Table 1, representing different connectivity parameter settings. The *t*-scores do not significantly correlate with either *W* or *f* across simulation models, demonstrating that GC computed from averaged voxel time series is not sensitive to true connectivity.

We then considered the effect of averaging the BOLD activity of all voxels in an ROI on the measurement of interregional GC. The GC between two ROIs, each of which is represented by an averaged time series, was measured by a single *t*-score in each direction (see Methods). For it to properly portray the connectivity between ROIs, the *t*-score was expected to follow the change of parameters across the simulation models shown in Table 1. We tested this prediction by measuring the correlation of the *t*-scores from the averaged time series with two summary statistics (the fraction of significant connections, *f*, and the average connectivity strength, *W*) (see Methods). These summary statistics were computed directly from the simulation models, and thus followed the change of parameters across the simulation models. The *t*-scores derived from averaged time series were not significantly correlated (*p*<0.05) with either summary statistic (Figure 2). That the *t*-scores did not follow the change of parameters across simulation models indicates that computing GC from averaged voxel time series does not accurately capture inter-ROI connectivity patterns.

**Figure 3:**
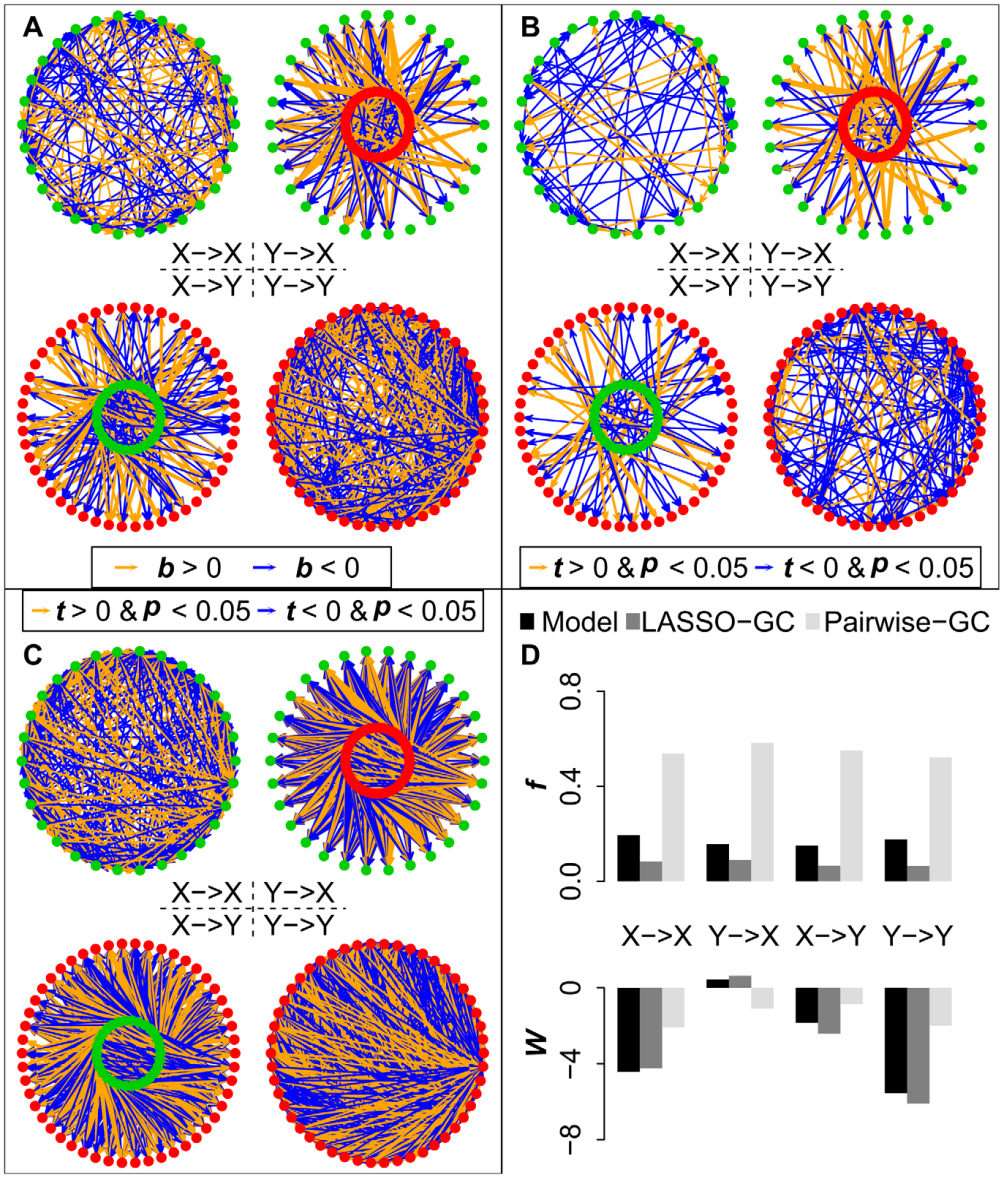
Comparison of model estimation by LASSO-GC and pairwise-GC methods for one simulation model. The X voxels in A-C are represented by green dots and Y voxels by red dots. All the *t* and *b* values are *z*-normalized. A) Simulated connectivity pattern of the model for the four B matrices, with orange arrows representing positive *b* values and blue arrows negative *b* values. B) Estimated connectivity pattern with LASSO-GC method. Significant *t*-scores are shown as arrows, with the thickness representing the absolute magnitude of the *t*-scores, and the color representing the sign of the *t*-score (orange for positive, blue for negative). The pattern is similar to that in the model. C) Estimated connectivity pattern with pairwise-GC method, shown in the same manner as for the LASSO-GC result. The connectivity is much denser than the model pattern. D) Summary statistics *f* and *W* for the patterns shown in the previous three panels. LASSO-GC values match the model values more closely than do pairwise-GC values.

We next examined how well voxel-based methods recovered the actual GC patterns of the four submatrices across the simulation models shown in Table 1. The analysis for each simulation model consisted first of estimating the full B matrix from the simulated data generated by that model using two methods: (1) pairwise-GC estimation; and (2) LASSO-GC estimation, i.e. LASSO pre-selection of variables for inclusion in an MVAR model, followed by GC estimation from the model. Then, the results from each method were compared with the actual values in the model. Each of these two methods is voxel-based. The pairwise-GC method constructs the B matrix by estimating a separate bivariate AR model for each voxel pair, whereas the LASSO-GC method computes the B matrix by estimating an MVAR model whose variables are pre-selected by LASSO. Unlike the approach of averaging across voxels, both methods compute a *t*-score for each *b* coefficient in the B matrix, testing whether the value of that coefficient significantly deviates from zero. A significant non-zero *b* value is equivalent to a significant GC value when the model order is one. Figure 3 illustrates the results from a simulation in which the LASSO-GC method (Figure 3B) closely estimated the pattern of *b* values of the model (Figure 3A), whereas the pairwise-GC method (Figure 3C) yielded a large number of spurious non-zero values.

**Figure 4:**
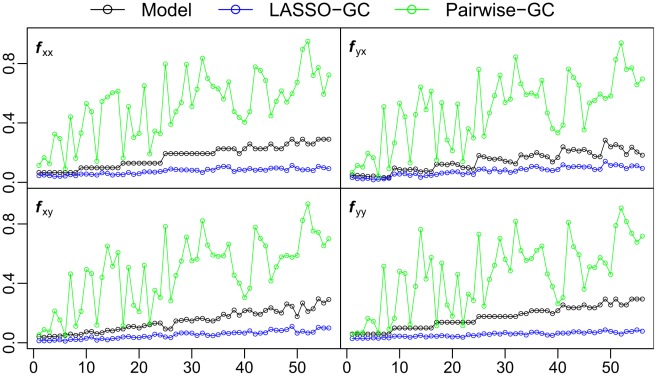
Comparison of LASSO-GC and pairwise-GC methods in recovering the *f* summary statistic. The fraction of significant *b* coefficients (*f* summary statistic) in each submatrix, computed directly from the simulation model, is compared with the *f* statistic estimated by the LASSO-GC and pairwise-GC methods. The estimated LASSO-GC *f* statistic more closely matches the *f* statistic of the model across simulation models than does the estimated pairwise-GC *f* statistic. The horizontal axis is arranged the same way as in Figure 2. The example shown in Figure 3 is from the 28th model.

To determine how typical were the results seen in Figure 3 across all simulation models, summary statistics from pairwise-GC and LASSO-GC estimations were compared with those computed directly from the models. First to be used was the *f* summary statistic, which measures the fraction of significant *b* values. Figure 4 compares how well the pairwise-GC and LASSO-GC methods recovered the actual *f* summary statistic computed directly from the simulation models. It reveals that in most simulations the *f* summary statistic from pairwise-GC estimation was greater than the actual simulation model value, whereas that from LASSO-GC estimation closely matched the actual simulation model value. We defined the distance between estimated and model *f* values by their absolute difference, and compared the distances resulting from the LASSO-GC method with that from the pairwise-GC method. Paired *t*-tests showed highly significantly (*p*<0.01) smaller distances with the LASSO-GC method for all four submatrices.

**Figure 5:**
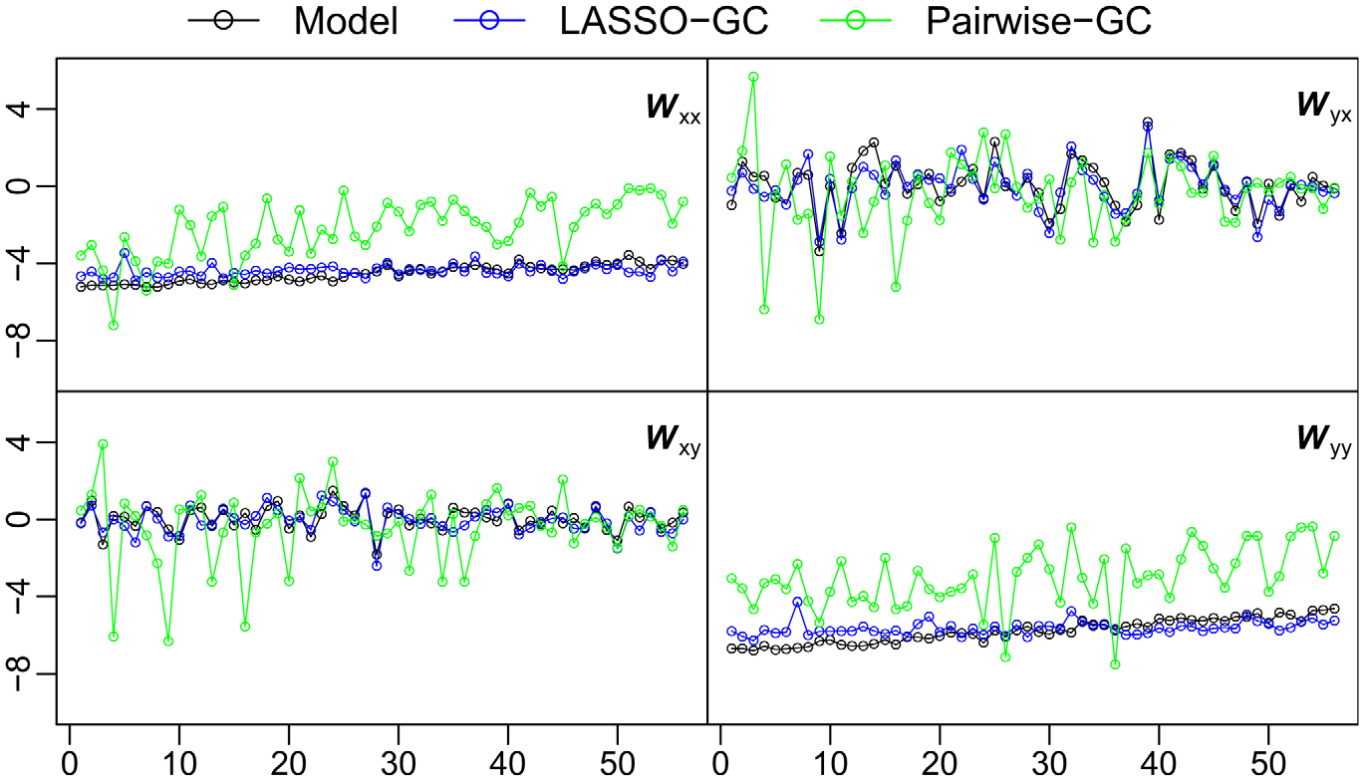
Comparison of LASSO-GC and pairwise-GC methods in recovering the *W* summary statistic. The GC strength (W summary statistic) in each submatrix, computed directly from the simulation model, is compared with the *W* statistic estimated by the LASSO-GC and pairwise-GC methods. The estimated LASSO-GC *W* statistic more closely matches the *W* statistic of the model across simulation models than does the estimated pairwise-GC *W* statistic. Since the estimated *W* statistic is based on *t*-scores and the *W* statistic computed directly from the simulation model is based on *b* coefficient values, both *b* and *t*-scores were normalized to standard *z*-scores before calculating *W*. The horizontal axis is arranged the same way as in Figure 2.

The *W* summary statistic, which reflects the average strength of significant GC from voxels in one ROI to voxels in another, was used next to compare the pairwise-GC and LASSO methods. As with the *f* statistic, the *W* statistic from the LASSO method matched the actual *W* statistic computed from the simulation model more closely than that from the pairwise-GC method (Figure 5). Also as with the *f* statistic, the distances between estimated and model *W* values for the two methods were compared. Paired *t*-tests showed highly significantly (*p*<0.01) smaller distances with the LASSO-GC method for all four submatrices.

The comparison of LASSO-GC versus pairwise-GC across 56 runs can be considered as 56 repeated tests of the two methods for their efficiency in estimating model parameters. The fact that LASSO-GC yielded more accurate estimations than pairwise-GC over a range of different parameter settings demonstrates LASSO-GC's robustness. To further validate this conclusion, we repeated each 56-run test on 20 separate iterations, each iteration using an independently generated dataset with the parameters from Table 1. The resulting GC patterns across the 20 iterations are consistent with those shown in Figures 2, 4 and 5 (as demonstrated in Figures S1, S2 and S3). To summarize the results up to this point, the LASSO-GC method was found to outperform the pairwise-GC method and the average-signal based method in recovering simulation model connectivity. We next applied the LASSO-GC method to explore functional connectivity in an empirical fMRI BOLD dataset.

### Application to fMRI BOLD data from a visuospatial attention task

An fMRI BOLD dataset from a slow event-related visuospatial attention task paradigm was analyzed with the LASSO-GC method. Details about the experimental design and the fMRI recording are available in [24] and [34]. Within each of 6 subjects, bilateral areas V1v, V2v, VP, V3A and V4 were in the Visual Occipital Cortex (VOC), and bilateral areas Frontal Eye Field (FEF) and anterior and posterior IntraParietal Sulcus (aIPS and pIPS) were in the Dorsal Attention Network (DAN). MVAR models of order-one were estimated from the time series of all voxels from each pair of VOC and DAN ROIs by the LASSO-GC method. The largest MVAR model contained approximately 150 voxels. Repeated trials (average number ∼70) at each time point were used as observations. For each ROI pair, a full B matrix was first estimated, and the *f* and *W* statistics were then computed for each of the four submatrices.

As with the simulation results, the correlations of the MVAR model residuals were found to be low, indicating that the models were valid. Because of the large data dimension, not all ROI pairs could be examined. Instead, 10 ROI pairs were randomly selected from each subject for examination: the residuals correlation matrix for one representative ROI pair is displayed in Figure S7. Since most of the correlation scores were weak (near or below 0.5), the MVAR models were considered to be valid representations of the fMRI BOLD data, and we thus proceeded to explore the connectivity patterns.

**Figure 6:**
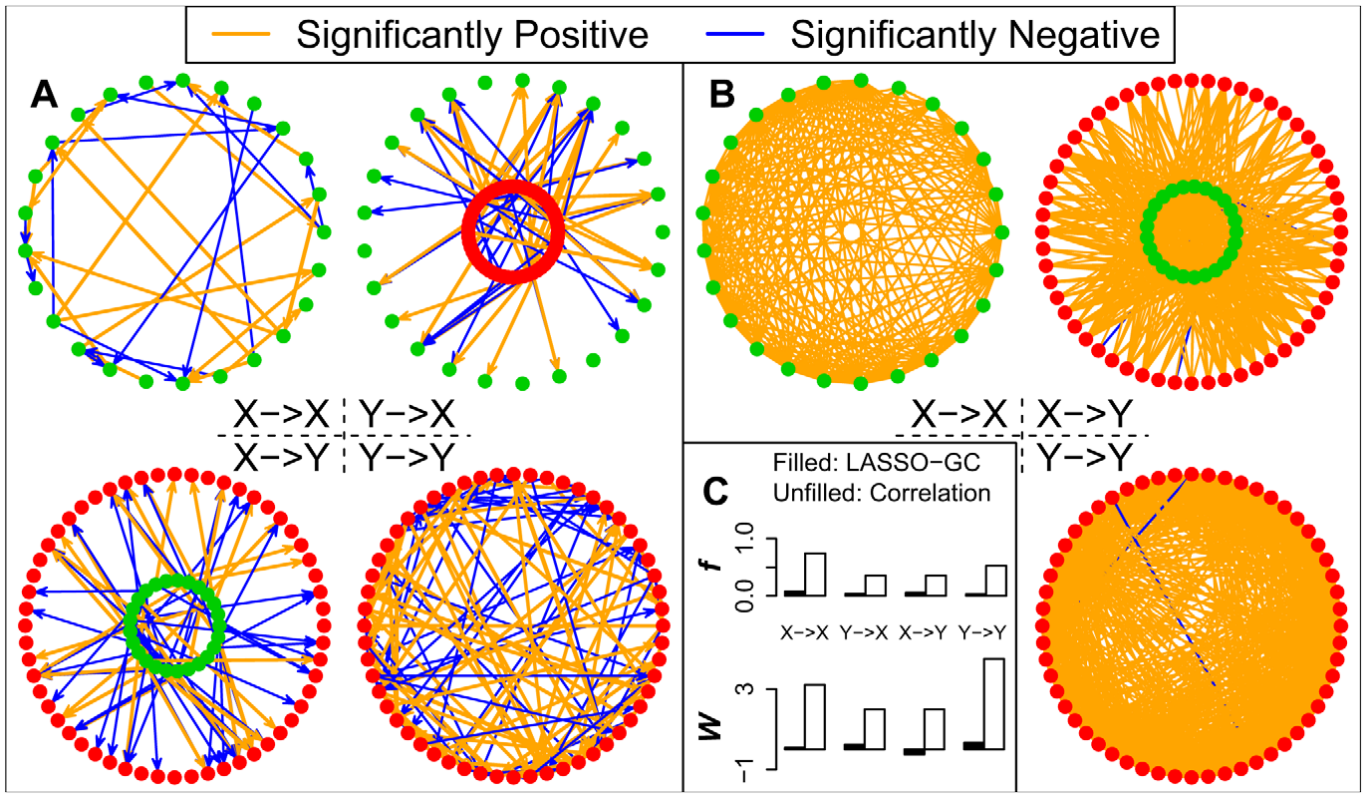
Comparison of connectivity patterns with LASSO-GC and correlation measures. Patterns are shown for one exemplary ROI pair from one subject. The *t*-scores from LASSO-GC analysis were z-normalized. Green dots represent voxels from right VP and red dots represent voxels from right FEF. A) Estimated connectivity patterns with the LASSO-GC measure. Significant *t*-scores are shown as arrows, with the thickness representing the absolute magnitude of the *t*-scores, and the color representing the sign of the *t*-score (orange for positive, blue for negative). B) Estimated connectivity patterns with the correlation measure. Significant cross-correlation coefficients are shown as lines, with the thickness representing the absolute magnitude and the color representing the sign (orange for positive, blue for negative). C) Summary statistics for the patterns shown in the previous two panels. For the correlation measure, FEF→VP and VP→FEF have the same summary scores since the measure is non-directional.

The results of functional connectivity analysis between the VOC and DAN are presented in Figure 6 for a representative ROI pair in one subject. GC connectivity diagrams are shown between the right VP region (having 25 voxels) in VOC and the right FEF region (having 56 voxels) in the DAN (Figure 6A). The four diagrams represent GC connectivity within right VP (VP → VP), from right FEF to right VP (FEF → VP), from right VP to right FEF (VP → FEF), and within right FEF (FEF → FEF). GC connectivity is sparse both within and between ROIs, meaning that a low fraction of *t*-scores is significant at *p*<0.05 (*f_VP−>VP_* = 0.08, *f_FEF−>VP_* = 0.04, *f_VP−>FEF_* = 0.06, *f_FEF−>FEF_* = 0.03). Both significantly positive (orange arrows) and significantly negative (blue arrows) GCs are present both within and between ROIs. A positive GC indicates that increased activity of the “sending” voxel predicts increased activity of the “receiving” voxel, whereas a negative GC signifies that increased activity of the “sending” voxel predicts decreased activity of the “receiving” voxel.

Connectivity based on the correlation measure is also considered. For correlations measured directly on the fMRI BOLD time series, a larger fraction of connections is significant at *p*<0.05 (*f_VP−VP_* = 0.67, *f_VP−FEF_* = 0.30, *f_FEF−FEF_* = 0.45) for the same ROI pair and subject (Figure 6B), suggesting that a large portion of the voxels are correlated. More sparse connectivity from LASSO-GC than from correlations is clearly seen in the *f* and *W* summary statistics (Figure 6C). This more sparse connectivity found with LASSO-GC than with correlation might have been a simple effect of LASSO pre-selection. However, this was found not to be the case since the correlations still showed much more dense patterns than the LASSO-GC results even when computed after LASSO pre-selection (Figures S4, S5). That the average correlation connectivity strength (*W* in Figure 6C) is greater than 2, both within and between ROIs, indicates that each voxel receives, on average, connections from more than 2 other voxels. This observation of relatively high correlation density suggests that the LASSO-GC method is needed to reduce correlation-induced spurious GC estimates.

**Figure 7:**
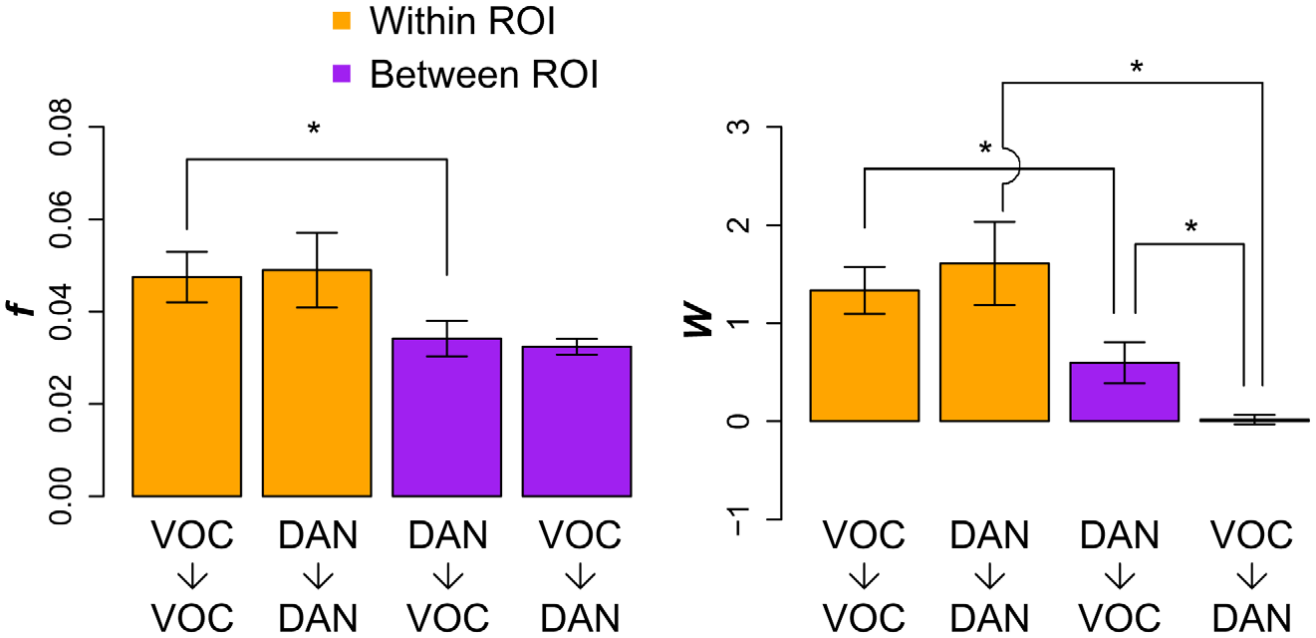
Functional connectivity analysis of Dorsal Attention Network and Visual Occipital Cortex in visual spatial attention. The *f* and *W* summary statistics were computed from LASSO-GC for each of 60 ROI pairs and 6 subjects, and then averaged over pairs and subjects. For each ROI pair, one ROI was in the Dorsal Attention Network (DAN) and the other was in Visual Occipital Cortex (VOC). The bars represent mean *f* and *W* summary statistics for VOC-to-VOC connectivity, DAN-to-DAN connectivity, DAN-to-VOC connectivity, and VOC-to-DAN connectivity. Error bars represent the standard error of the mean. Significant differences from paired-sample *t*-tests are marked (*: *p*<0.05).

To extend the functional connectivity analysis to the full fMRI BOLD dataset, we applied the LASSO-GC method to all 60 VOC-DAN ROI pairs in each of the 6 subjects. The *f* and *W* summary statistics were then averaged across ROI pairs and subjects, yielding mean *f* and *W* summary statistics for VOC-to-VOC connectivity, DAN-to-DAN connectivity, DAN-to-VOC connectivity, and VOC-to-DAN connectivity (Figure 7). These four connectivity types correspond to the four coefficient submatrices of the estimated B matrix in LASSO-GC analysis (see Methods): VOC-to-VOC and DAN-to-DAN connectivity refers to connectivity within a single region of VOC or DAN, not to connectivity between different VOC or DAN regions. The mean *f* summary statistic is below 0.1 for all submatrices, indicating overall sparse within- and between-ROI GC connectivity.

Paired-sample t-tests with subjects as repeated measures (df = 5 for all comparisons) were performed on both *f* and *W* to compare: (1) top-down (DAN-to-VOC) with bottom-up (VOC-to-DAN) connectivity; (2) within-VOC with within-DAN connectivity; (3) top-down with within-VOC connectivity; and (4) bottom-up with within-DAN connectivity. The comparison of top-down with within-DAN connectivity and the comparison of bottom-up with within-VOC connectivity were not performed because these comparisons are ambiguous, i.e., they are based on GC to voxels in a sending region, whereas the *W* summary statistic is based on voxels in a receiving region (see Methods). The results show that within-VOC (VOC→VOC) connectivity was significantly greater than top-down (DAN→VOC) connectivity for both the *f* (*t* = 4.60, *p*<0.05) and *W* (*t* = 2.85, *p*<0.05) summary statistics, indicating that the local GC between voxels within VOC is both more dense and stronger than the long-range, top-down GC from the DAN. Connectivity within DAN (DAN→DAN) was also significantly greater than that in the bottom-up direction (VOC-to-DAN) for the *W* summary statistic (*t* = 4.07, *p*<0.05), but not for the *f* summary statistic, indicating that the local GC between voxels within DAN is stronger, but not more dense, than the long-range GC from VOC. Finally, connectivity in the top-down direction (DAN→VOC) was significantly greater than that in the bottom-up direction (VOC→DAN) for the *W* summary statistic (*t* = 3.93, *p*<0.05) but not for the *f* summary statistic, indicating a long-range directional strength asymmetry between DAN and VOC, with stronger top-down connectivity.

## Discussion

We have shown that Granger Causality (GC) computed from voxel-level BOLD signals better reflects the pattern of directed functional interaction between ROIs than that computed from voxel-averaged signals. We conclude that brain regions are not unitary elements, that network structure exists at the voxel level, and that ROI-level GC connectivity is best measured by summary scores computed over voxel-level connectivity patterns.

We emphasize that our conclusions apply specifically to GC between pre-defined ROIs, and do not necessarily extend to the computation of maps showing GC between a “seed” signal, averaged over the voxels in one cortical region, and voxels throughout the rest of the cortex [35]. In fact, an interesting extension to the mapping approach has recently been proposed by Garg et al. [33]. Their technique, called Full-brain AutoRegressive Modeling (FARM), also adopts LASSO for voxel-level analysis. Although their use of LASSO to make MVAR modeling feasible for large numbers of voxels is similar to ours, their problem of GC mapping for the entire brain is different from the interregional interaction question that we have investigated. It is possible that full-brain mapping and interregional analysis will prove complementary to each other. To explore the relationship of a particular region to the remainder of the cortex, the mapping method would appear to be more appropriate since it yields a global functional interaction pattern. However, to test specific theories in cognitive neuroscience that involve particular cortical networks, might require the use of regions that are pre-defined from previous clinical or experimental evidence. In that case, one would be interested in examining the details of functional interaction between regions, and a full-brain LASSO algorithm could be insufficient because its tuning parameter might be too severe, making the model overly sparse: even if the global connectivity pattern were preserved, the details of interregional connectivity might still be lost. With these concerns in mind, one might use full-brain mapping as a first step to establish global functional interaction patterns, and then a more detailed exploration could be performed using voxel-based interregional analysis.

In addition to mapping, another common analytic method in the literature examines region-to-region correlations based on averaged signals and identifies topological properties from large-scale networks that involve hundreds of ROIs [36]. Our findings do not necessarily negate this approach: since GC and correlation are different measures, inhomogeneity in GC does not imply inhomogeneity in correlation. It is possible that correlation-based connectivity with averaged signals may be effective even though GC analysis requires a voxel-based approach.

We have demonstrated that the LASSO-GC method can better identify GC connectivity between ROIs in simulated fMRI BOLD data than the pairwise-GC method by more accurately estimating the connectivity density and strength. The pairwise-GC method can yield spuriously significant coefficients if correlated predictors are present in the MVAR model. The close fit of the LASSO-GC results to the actual results from the simulation models demonstrates that the LASSO-GC method is better able to avoid false positives, and also shows the sensitivity of this method in detecting model changes. By contrast, GC values computed from averaged data do not systematically follow changes in simulated ROI models, suggesting that summary statistics computed from voxel-to-voxel GCs are better able to represent ROI-level connectivity than single region-to-region GCs computed after averaging over ROI voxels.

The estimated *f* summary statistics from the LASSO-GC method matched the actual *f* statistics from the simulation models better when the B matrices were more sparse. Although the LASSO algorithm could potentially fail for high connectivity densities, we were not able to observe such a failure because the simulated voxel activity at high connectivity density becomes unstable. Nonetheless, it is unlikely that the low *f* values observed for the empirical BOLD data are artifactual because if the B matrices were ill-estimated, then the directional asymmetry found with the *W* statistic would not display the high degree of consistency across subjects that was observed. The fact that the range of *f* found for the empirical BOLD data fell within the range of *f* in the simulations further suggests the suitability of the LASSO-GC technique for application to BOLD data. Moreover, the low values of the *f* summary statistic from the empirical BOLD data indicate that GC connectivity between cortical ROIs is sparse. Given evidence from anatomical studies that axonal connectivity of the cortex is generally sparse [37], [38], it is more likely that the sparse GC connectivity reflects actual functional interaction patterns than that it is a mere statistical byproduct.

Directional asymmetry in GC connectivity between the Dorsal Attention Network (DAN) and Visual Occipital Cortex (VOC) was reported in our previous work [24] using the pairwise-GC method for computing GC and *f* as the summary statistic. Using the LASSO-GC method, we report here that the directional asymmetry is found in the *W*, but not the *f*, summary statistic (Figure 5). The difference in results from the pairwise-GC and LASSO-GC methods may be understood by examining the properties of the *W* summary statistic. The finding that *W* values in the top-down DAN-to-VOC direction are significantly greater than in the bottom-up VOC-to-DAN direction suggests that VOC voxels are modulated more strongly by DAN voxels than DAN voxels are by VOC voxels, despite there being similar fractions of voxels being modulated in both directions. The greater top-down modulation strength may have introduced a bias in the pairwise-GC results from our previous work, yielding an apparently greater fraction of significant top-down GC values. Relatively high correlation density (Figure 6) may have contributed to such a bias.

The problem of bias actually has multiple facets. It is known from theory that the LASSO method may be biased if predictors are highly correlated. There are two main problems caused by correlated predictors. First, some predictors in a system may not be included in the model of the system. This is the case when estimation of multiple bivariate AR models is employed in place of MVAR model estimation: the estimation may be biased by undetected influences from the excluded predictors. The use of LASSO helps to mitigate this problem by allowing estimation of a full MVAR model. Second, even when all the predictors are taken into account, correlation among predictors may still bias model estimation, a situation often referred to as the collinearity problem for multiple regressions. An example of such bias would be the case of a group of strongly correlated predictors, where LASSO tended to select only one predictor from the group. Extensions of LASSO have been proposed to mitigate this problem by selecting the entire group instead of a single predictor. Such extensions include fused LASSO [39], the elastic net [40] and the group LASSO [41]. A thorough review of this issue is available in [42]. Whether such selection bias becomes a problem in brain network analysis depends on the specific research question being considered. On the one hand, if the exact relationship among predictors is of central interest, as when large-scale cortical network structure is explored using ROIs as predictors [28], one may consider the use of extended LASSO algorithms to avoid losing important correlated ROIs. On the other hand, in our voxel-based investigation of pre-selected ROIs, the voxels are used as multiple representations of the corresponding ROIs, and omitting some of the correlated voxels in a group is not expected to radically change the collective functional connectivity at the ROI level. In the present study, we are more interested in the summary statistics over the entire connectivity matrix than in the details of the connectivity patterns within the matrix. Thus, in our study, although the collinearity problem may exist, and a complete solution is not currently available from theory, our results are nonetheless not invalidated. We found that the *f* and *W* summary statistics effectively recovered the modeled connectivity values from simulated data, even though those data had significant correlations between most voxel pairs. Furthermore, in empirical BOLD data analysis, it is often desirable to compare summary statistics across different conditions rather than to precisely identify their values. For such comparison, any possible bias introduced by voxel-voxel correlations would exist in both conditions and thus would not alter the comparison.

Although the MVAR models used in this paper were implemented with order one, models having higher order (*p*>1 in Equation 3) can be implemented within the same framework. For model orders greater than one, multiple *b* coefficients at different time lags (t-k) contribute to the GC from one voxel to another, and it is not sufficient simply to test the significance of a single *b* coefficient. In that case, testing for significant between-voxel GC would need to be performed differently, and the summary statistics would accordingly be defined differently. For example, a criterion for between-voxel GC to be significant might be that at least one of the *b* coefficients from different lags must be significant. A summary statistic equivalent to *f* might then be defined as the fraction of significant between-voxel GCs rather than the fraction of significant *b* values. Similarly, a summary statistic equivalent to *W* could base the average strength of significant GC on all significant *b* values for a voxel pair instead of a single *b* value. A straightforward way to do this would be to sum the significant *b* values from different lags over all inputs to receiving voxels. In this way the *W* statistic would be sensitive to three factors: the magnitude of all significant *b* values, their corresponding time lags, and the total number of converging significant inputs to receiving voxels. However, to compare the *W* statistic between models of different order, the time-lag factor would need to be removed, possibly by averaging *b* values over time lags, to avoid bias due to different total numbers of *b* values.

In conclusion, our work suggests that the LASSO algorithm can be effectively employed for pre-selection of voxels that are then used in an MVAR model to measure functional connectivity between ROIs, using voxel-based fMRI BOLD signals. It indicates that the *f* and *W* summary statistics reveal different aspects of directed influence between ROIs. Used in tandem, these statistics may provide consistent information about influences between brain regions that is richer than that from either one alone. Additional summary statistics will likely be found in the future that will further our understanding of directed influences between brain regions.

## Methods

### The MultiVariate AutoRegressive (MVAR) model

We first consider an fMRI BOLD dataset from *m* voxels in ROI X and *n* voxels in ROI Y. The dataset consists of time series of *t* points recorded from every voxel in X and Y, and can be written in matrix form as:
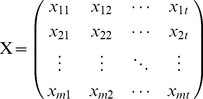
(1)

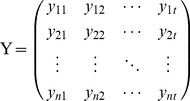
(2)


The relationship between X and Y can be expressed in the form of a Multivariate Vector AutoRegressive (MVAR) model. A general matrix representation of the model is:
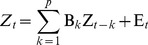
(3)where Z*_t_* is the dependent variable in vector form, representing the BOLD data values at arbitrary time *t* of all voxels in X and Y; Z*_t-k_* represents the values of the Z vector at arbitrary earlier time point *t*-*k*; lag *k* ranges from 1 to *p*, the model order; B*_k_* is the corresponding coefficient matrix at lag *k*; and E*_t_* is the residual vector.

When expanded, the product term in Equation 3 becomes:
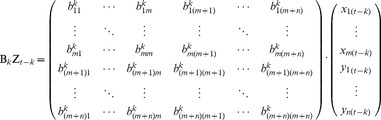
(4)


Each element of the Z*_t-k_th* vector is a predictor, and each element (*b^k^_ij_*) of the B*_k_* matrix is a coefficient representing the degree of prediction of the *i*th element of Z*_t_* by the *j*th predictor. If a value of *b^k^_ij_* significantly differs from zero, then a significant GC is said to exist from voxel *j* to voxel *i*. The magnitude (strength) of that GC may be assessed by the magnitude of the statistic (e.g. *t*-statistic) used to measure the difference of the *b* value from zero. The sum of product terms over all lags is the total prediction of Z*_t_* by the model.

The model order (*p*) was set to one in this paper, based on our prior experience with the analysis of fMRI BOLD data [24]. The MVAR model in Equation 3, with model order one, was used here for both simulation and GC analysis. For simulation, the residual vector represented an innovation process that generates random values, the B matrix was known, and the X and Y time series data were simulated. For GC analysis, the X and Y time series data were known, the B matrix was estimated in order to determine GC, and the residual vector represented prediction errors.

We also employed pairwise-GC analysis for comparison with MVAR analysis. In the pairwise-GC approach, coefficients are estimated (and the significance of GC determined) by constructing a separate bivariate model for each pair of voxels, one in X and one in Y:
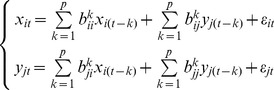
(5)


In pairwise-GC analysis, the assumption is made that the predictors are independent of one another. Under this assumption, the GC between X and Y can be assessed solely from the bivariate models in Equation 5, and it is not necessary to estimate the coefficients representing GC within X or Y. In fact, however, the predictors may be correlated for BOLD time series, making the pairwise-GC approach problematic. If the predictors are correlated, estimation by separate bivariate (or partial) models may be biased, and all of the coefficients in the B matrix should be estimated simultaneously [43]. Nonetheless, simultaneous estimation may be impossible in the analysis of data from neurobehavioral studies, in which the number of observations is often limited.

### The Least Absolute Shrinkage and Selection Operator (LASSO)

The Least Absolute Shrinkage and Selection Operator (LASSO) technique is a method that makes model estimation feasible when only a limited number of observations is available. Under the assumption that the B matrix is sparse (i.e., many coefficients are zero), the LASSO algorithm effectively determines which *b* values are actually zero. Our goal in using LASSO is to identify non-zero coefficients and then estimate them simultaneously, thus avoiding bias due to partial regression with correlated predictors. The pre-selection process in LASSO involves determining an optimal set of predictors.

In the MVAR model, pre-selection is carried out in a row-wise manner. LASSO adds a constraint on each row equation of Equation 3 that restricts the total absolute values of the coefficients. The constraint is expressed as:
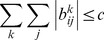
(6)where *c* is a tuning parameter.

Regression of the *i*th row of Equation 3 under the constraint provided by Equation 6 is equivalent to the regression of:
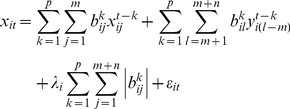
(7)


Finding a least-squares solution of Equation 7 requires a subset of the *b* values to be set to zero. To achieve this goal we use the Least Angle RegreSsion (LARS) algorithm developed by Efron et al. [44], which starts with all *b*'s equal to zero and then iteratively adjusts their values to fit the model. Some of the *b*'s remain zero after the adjustment, resulting in the identification of an optimal set of non-zero *b* values for a particular *c* value (corresponding to λ in Equation 7).

### The General Cross-Validation (GCV) criterion for determining optimal predictors

The next step in model estimation is to tune the parameter *c* to achieve a best fit of Equation 7. The minimum value that *c* can take is zero, corresponding to the extreme case where all *b*'s are zero. The upper boundary is reached when LARS does not penalize any *b* to zero, making *c* equal to the sum of the absolute values of all *b*'s. Within this interval, a number (approximately 100 in our case) of *c* values is chosen to compute the subsets of *b*'s. For each *c* value, after an optimal subset has been found, the corresponding Residual Sum of Squares (RSS) is used to calculate a General Cross-Validation (GCV) statistic [28]:
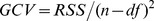
(8)where *n* is the number of independent observations and *df* is the estimated degrees of freedom from the LARS algorithm. From all the solutions, a GCV curve is plotted. The minimum GCV value determines the single most optimal set of predictors over all *c* values.

A subsequent Ordinary Least Squares (OLS) procedure is then applied to the new row equation with the selected predictors. To avoid using the same data to estimate the LASSO and OLS models, we randomly sort the data trials into two sets. One set is used to estimate the LASSO coefficients for model selection, and the other is then used to estimate OLS coefficients for the new row equation. In the second step, if the model order is one, as in our application, there is only one coefficient for each predictor. Either an *F*-test or a *t*-test is performed for each coefficient to determine whether its value is significantly different from zero. The resulting *F*-score or *t*-score characterizes the prediction by a predictor on the RHS of Equation 3 of the dependent variable on the LHS, and corresponds to the GC strength from that predictor to the dependent variable. Here we used the *t*-score to measure GC because it has a signed value, and thus indicates whether the GC is enhancing or reducing, in addition to indicating GC strength.

### Summary statistics of GC between two ROIs

The full B matrix may be estimated by following the above procedures for every row equation in Equation 3. It consists of four submatrices (B_xy_, B_yx_, B_xx_, and B_yy_), where the first subscripted index represents the predictor and the second represents the dependent variable. Thus, B_xy_ represents connectivity from X to Y, B_yx_ represents connectivity from Y to X, and B_xx_ and B_yy_ represent connectivity within X and within Y, respectively. In order to measure GC from one ROI to another (i.e., X→Y or Y→X), one or more statistics are needed to summarize the voxel-to-voxel GCs represented by significant coefficients in B_xy_ or B_yx_.

**Figure 8:**
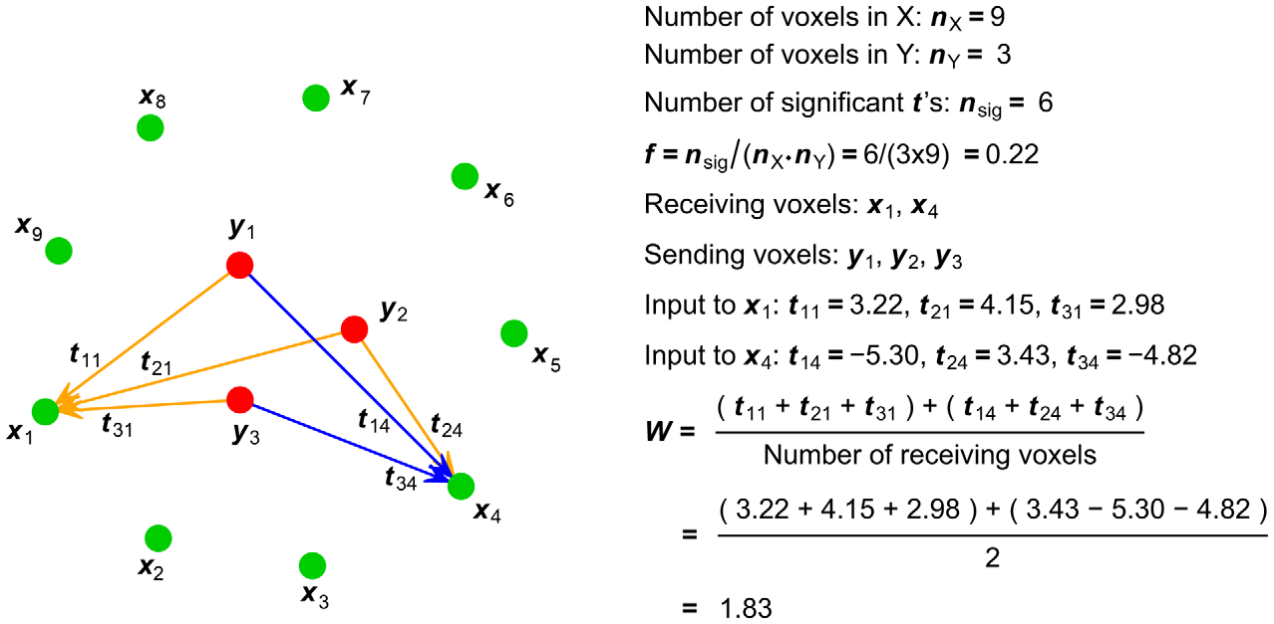
Schematic illustration of the computation of summary statistics *f* and *W* for hypothetical submatrix B_yx_. Red dots represent the voxels of ROI Y, green dots the voxels of ROI X, and arrows the significant *t*-scores between interregional voxel pairs. Positive values are colored orange and negative values are colored blue.

The first summary statistic that we used was the fraction (*f*) of significant *b* values in the B matrix or one of its submatrices (representing the fraction of significant GCs) (Figure 8). The fraction of *b* values found to be significantly different from zero at p<0.05 was corrected for multiple-comparisons by the False Discovery Rate (FDR). This summary statistic is a measure of density of the ROI-level connectivity. Because each *b* value represents a potential functional “connection”, the *f* summary statistic summarizes the fraction of all possible voxel-to-voxel connections from one ROI to another by which the two ROIs are actually connected.

The second summary statistic used was the average strength of significant GC from voxels in one ROI to voxels in another (Figure 8). Consider, for example, the GC from (“sending”) ROI Y to (“receiving”) ROI X. Significant voxel-to-voxel GCs are represented by significant coefficients in B_yx_. For any given voxel *x* in ROI X having at least one significant (*p*<0.05) *t*-score (indicating a significant GC) from ROI Y, we first summed the *t*-scores of all the GCs to *x*. This sum represents the total significant “input” to the “receiving” voxel *x* from all “sending” voxels in Y. Because the *t*-scores can be positive or negative, signifying that changes of activity in the “sending” voxel contribute to a change of activity in the “receiving” voxel either in the same or opposite direction, the sum of *t*-scores takes into account the balancing effect of positive and negative inputs to the same receiving voxel. We then computed the average strength of significant input over all receiving voxels in ROI X as the *W* summary statistic. The significance threshold for determining the number of significant inputs was corrected for multiple-comparisons by the FDR. The same procedure was also followed to assess the average strength of significant GC in the other direction, i.e. from ROI X to ROI Y using B_xy_. Although not the focus of this paper, *W* could also be computed to assess the average strength of significant GC within ROI X using B_xx_, or within ROI Y using B_yy_. *W* measures the average strength of GC from ROI Y to ROI X, but is not simply a weighted version of the *f* summary statistic. For example, a high *W* value from Y to X depends on a combination of the following: 1) many voxels in Y have high GC values to voxels in X; and 2) single voxels in X have significant GC values from multiple voxels in Y.

Simulation models were constructed using the R statistical computing package. For the purpose of comparing the GC strength of a simulation model with its estimated values, we computed the simulation *W* statistic directly from the *b* values of the simulation model. To make the estimated and simulation *W* measures comparable, we normalized the *t* and *b* values to *z*-scores (i.e. subtracted the mean and then divided by the standard deviation). Computer code used in this study will be freely provided for legitimate research purposes upon request from the first author.

## Supporting Information

Figure S1 **Granger Causality patterns between simulated ROIs with multiple iterations.** Comparison of *t*-scores from the averaging approach and the voxel-based *f* and *W* summary statistics computed directly from the model parameters, as in Figure 2 but with multiple iterations of each parameter set. Each parameter set was repeatedly simulated with 20 iterations. Vertical bars show the standard error across runs.
(TIF)

Figure S2 **Comparison of LASSO-GC and pairwise-GC methods in recovering the** ***f*** **summary statistic.** Same comparison as in Figure 4, but with multiple iterations of each parameter set. Each parameter set was repeatedly simulated with 20 iterations. Vertical bars show the standard error across runs.
(TIF)

Figure S3 **Comparison of LASSO-GC and pairwise-GC methods in recovering the** ***W*** **summary statistic.** Same comparison as in Figure 5, but with multiple iterations of each parameter set. Each parameter set was repeatedly simulated with 20 iterations. Vertical bars show the standard error across runs.
(TIF)

Figure S4 **Estimated connectivity patterns with the correlation measure.** After the LASSO procedure, some of the coefficients in the connectivity matrix of the MVAR model were set to zero. The correlation scores were then computed for the voxel pairs having non-zero coefficients. Since both GC and correlation measures were computed after the LASSO procedure, they could be compared without the possibility of a connectivity bias due to LASSO. The display scheme is the same as in Figure 6B.
(TIF)

Figure S5 **Comparison of the** ***f*** **summary scores.** The *f* summary score is compared for measures of correlation without LASSO (blue), correlation with LASSO (red), and LASSO-GC (yellow). Supplementary to Figure 6C.
(TIF)

Figure S6 **The model residuals correlation matrix for one simulation run.** Each cell represents a color-coded correlation score between model residuals from two simulated voxels in the MVAR model. The diagonal cells represent the correlation of the voxel with itself, which always equals 1.
(TIF)

Figure S7 **The model residuals correlation matrix for a representative ROI pair.** Each cell represents a color-coded correlation score between model residuals from two voxels in the MVAR model estimated from the fMRI BOLD data. The diagonal cells represent correlation of the voxel with itself, which always equals 1.
(TIF)
