# Supplementary figures and images for: Measuring Granger Causality between Cortical Regions from Voxelwise fMRI BOLD Signals with LASSO
Source: PLoS Comput Biol. 2012 May 24;8(5):e1002513. doi: 10.1371/journal.pcbi.1002513 (PMC3359965; doi:10.1371/journal.pcbi.1002513)

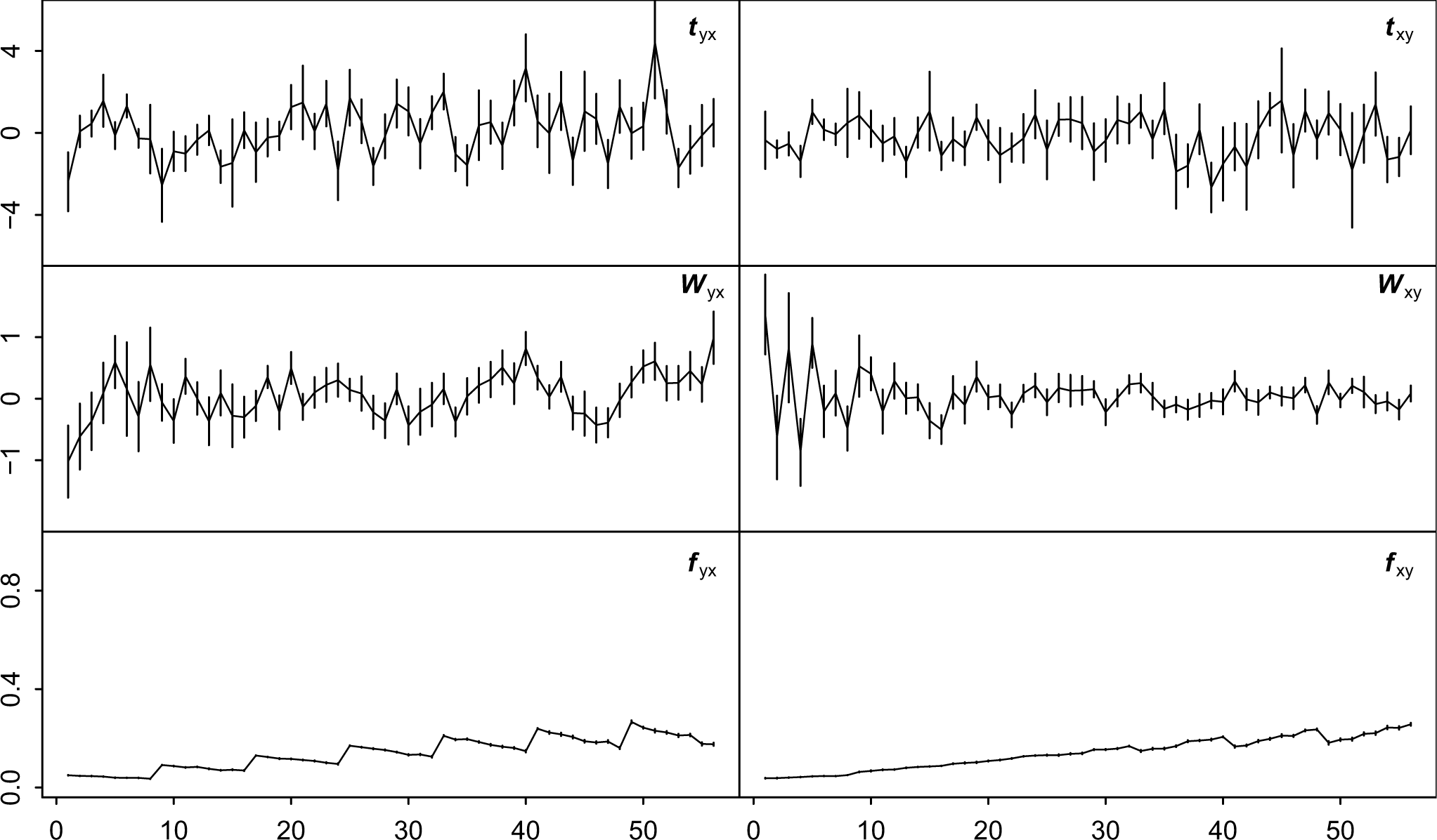

Supplement: Figure S1 — Granger Causality patterns between simulated ROIs with multiple iterations. Comparison of t-scores from the averaging approach and the voxel-based f and W summary statistics computed directly from the model parameters, as in Figure 2 but with multiple iterations of each parameter set. Each parameter set was repeatedly simulated with 20 iterations. Vertical bars show the standard error across runs. (TIF) [file pcbi.1002513.s001.tif]

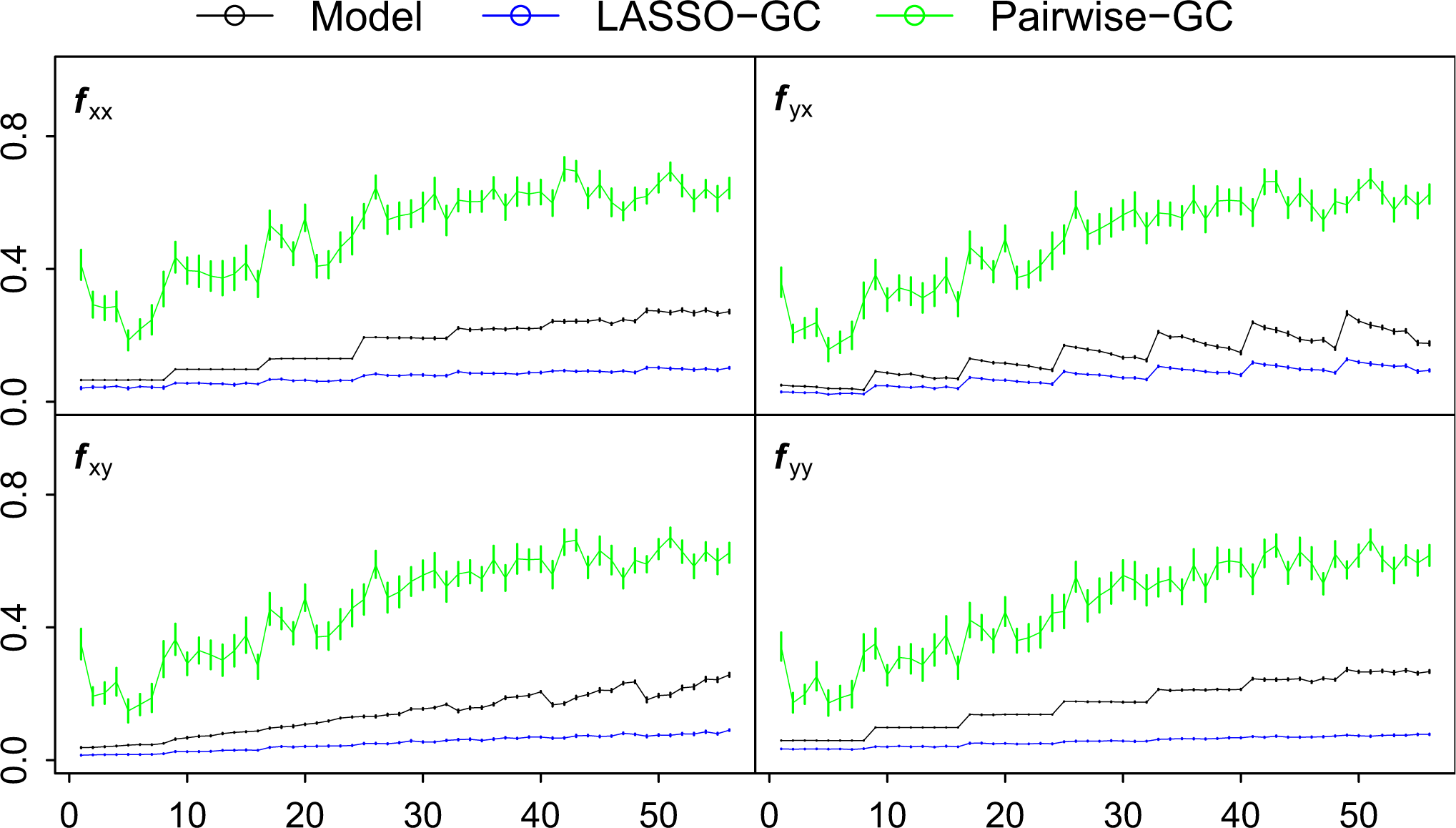

Supplement: Figure S2 — Comparison of LASSO-GC and pairwise-GC methods in recovering the f summary statistic. Same comparison as in Figure 4, but with multiple iterations of each parameter set. Each parameter set was repeatedly simulated with 20 iterations. Vertical bars show the standard error across runs. (TIF) [file pcbi.1002513.s002.tif]

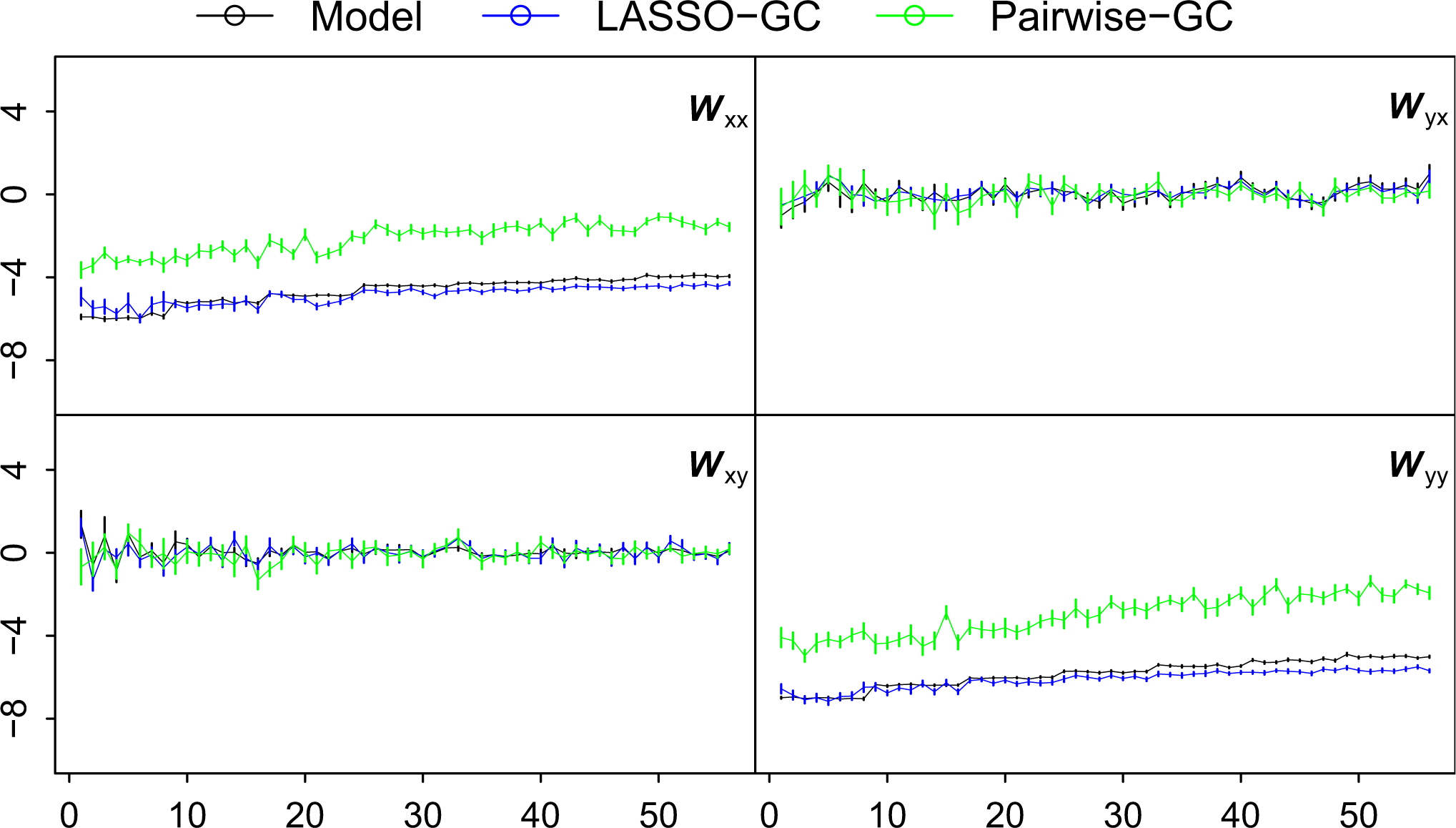

Supplement: Figure S3 — Comparison of LASSO-GC and pairwise-GC methods in recovering the W summary statistic. Same comparison as in Figure 5, but with multiple iterations of each parameter set. Each parameter set was repeatedly simulated with 20 iterations. Vertical bars show the standard error across runs. (TIF) [file pcbi.1002513.s003.tif]

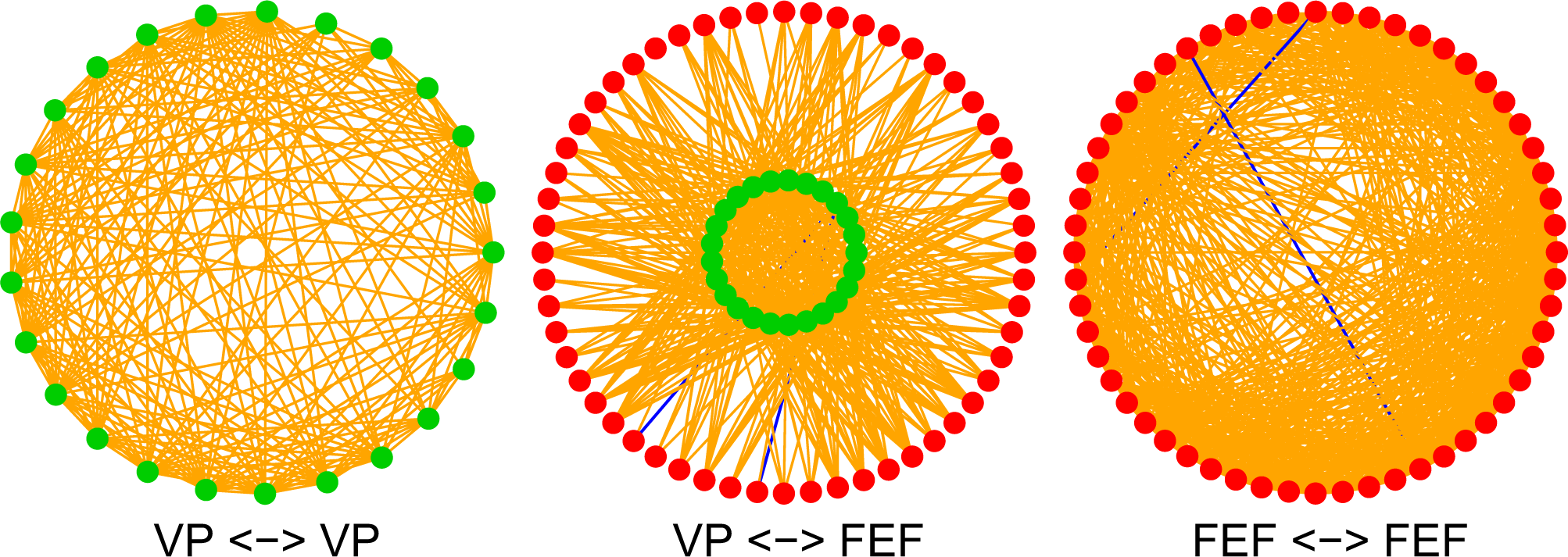

Supplement: Figure S4 — Estimated connectivity patterns with the correlation measure. After the LASSO procedure, some of the coefficients in the connectivity matrix of the MVAR model were set to zero. The correlation scores were then computed for the voxel pairs having non-zero coefficients. Since both GC and correlation measures were computed after the LASSO procedure, they could be compared without the possibility of a connectivity bias due to LASSO. The display scheme is the same as in Figure 6B. (TIF) [file pcbi.1002513.s004.tif]

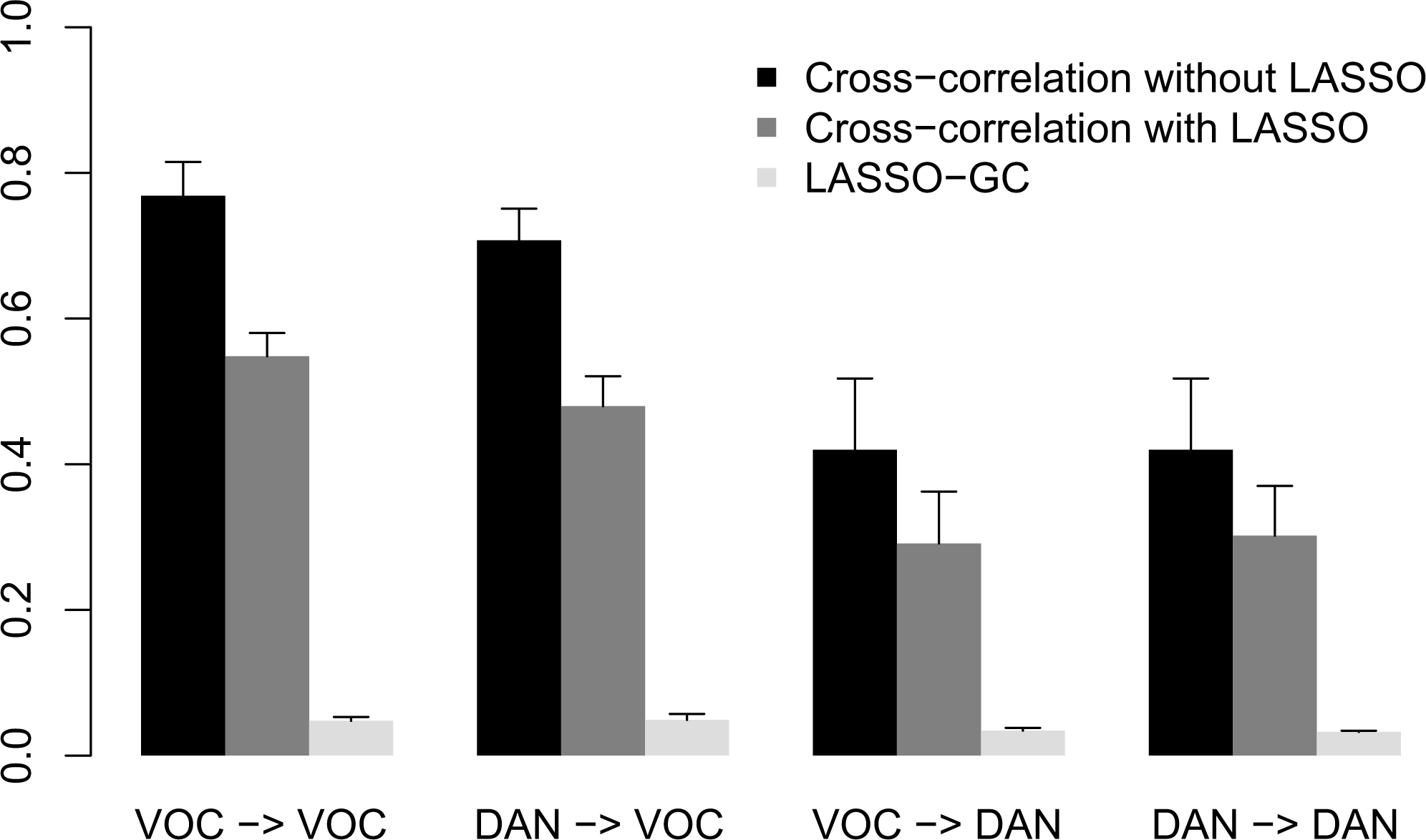

Supplement: Figure S5 — Comparison of the f summary scores. The f summary score is compared for measures of correlation without LASSO (blue), correlation with LASSO (red), and LASSO-GC (yellow). Supplementary to Figure 6C. (TIF) [file pcbi.1002513.s005.tif]

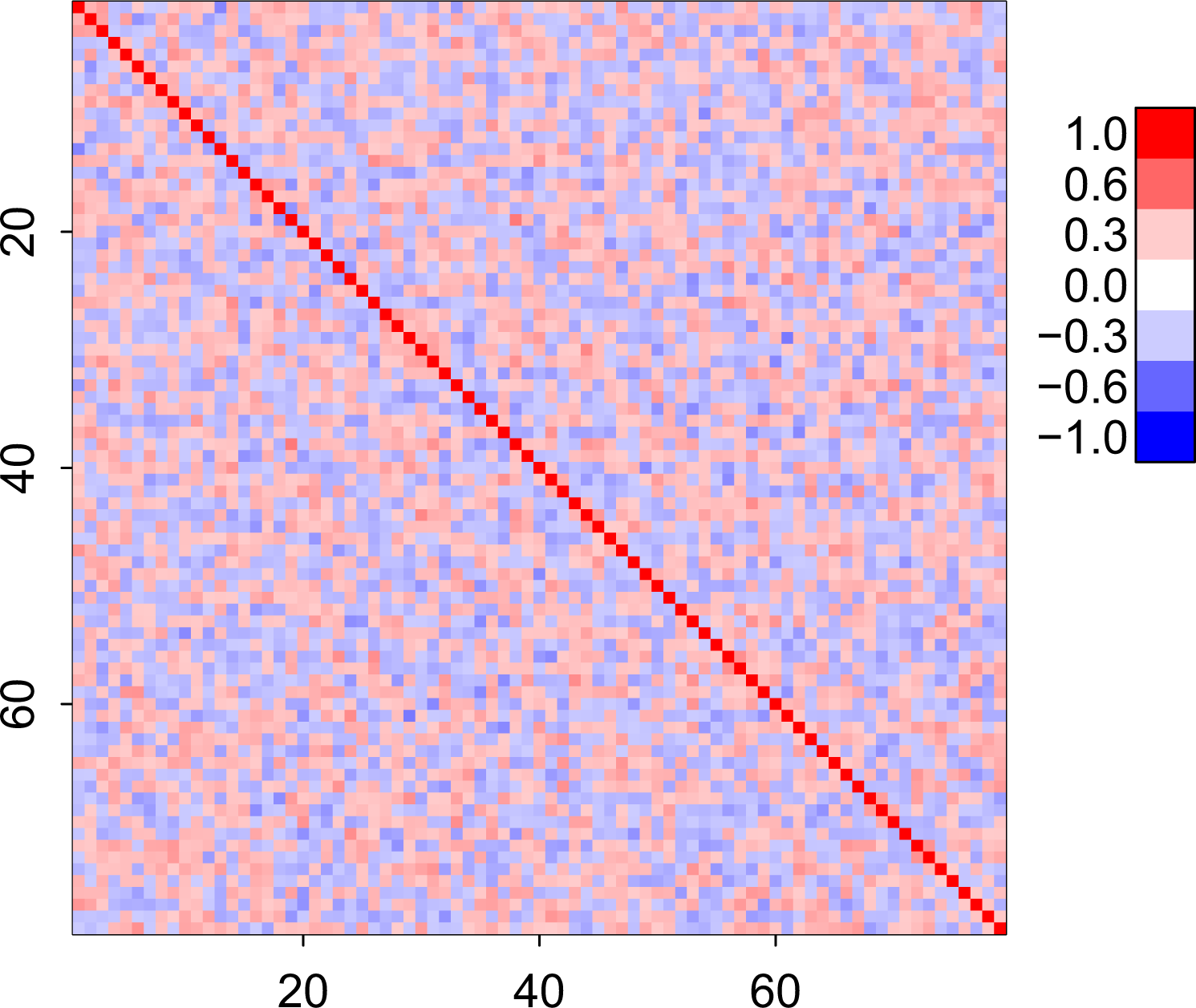

Supplement: Figure S6 — The model residuals correlation matrix for one simulation run. Each cell represents a color-coded correlation score between model residuals from two simulated voxels in the MVAR model. The diagonal cells represent the correlation of the voxel with itself, which always equals 1. (TIF) [file pcbi.1002513.s006.tif]

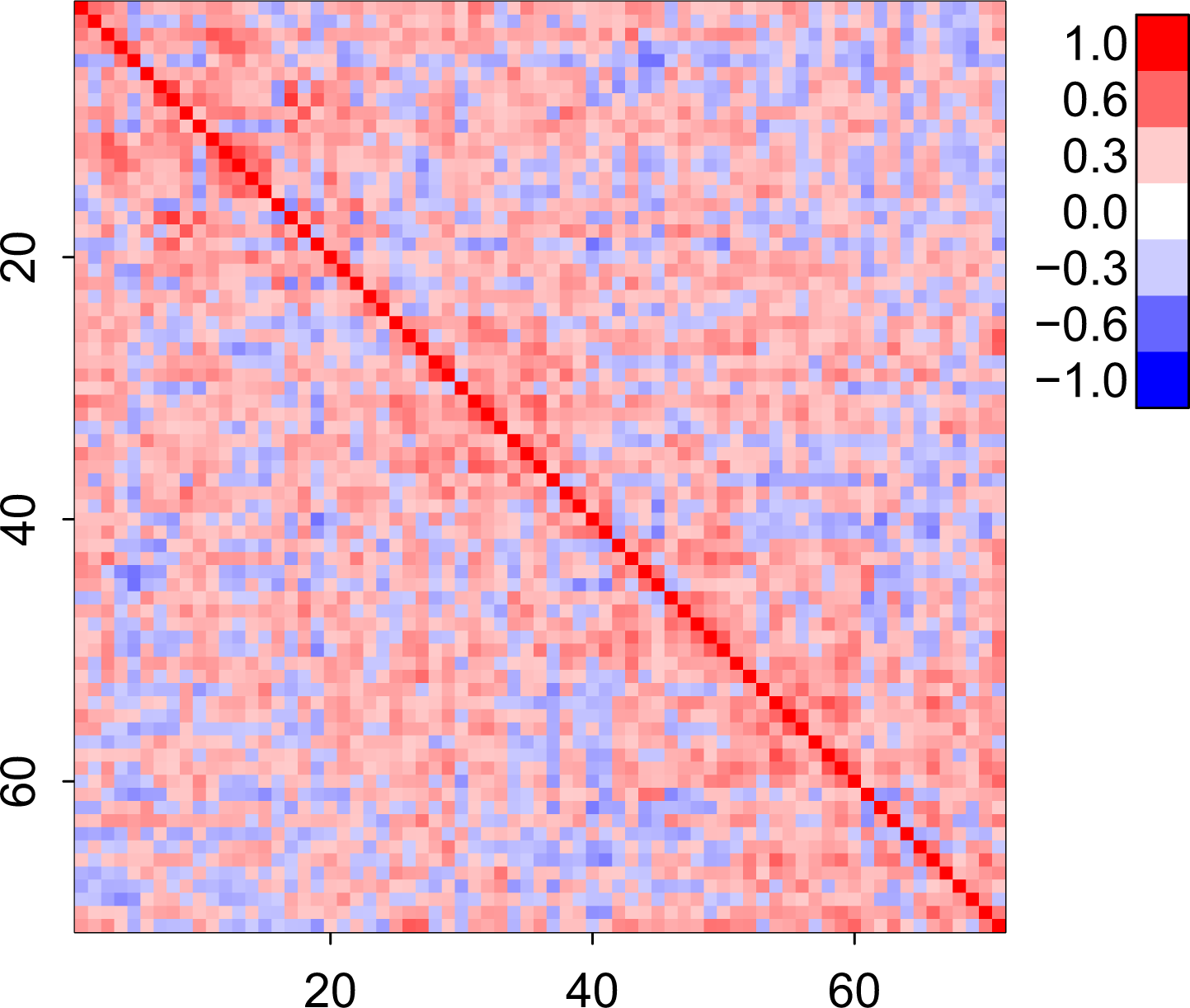

Supplement: Figure S7 — The model residuals correlation matrix for a representative ROI pair. Each cell represents a color-coded correlation score between model residuals from two voxels in the MVAR model estimated from the fMRI BOLD data. The diagonal cells represent correlation of the voxel with itself, which always equals 1. (TIF) [file pcbi.1002513.s007.tif]
